# The Essential Need for Research Misconduct Allegation Audits

**DOI:** 10.1007/s11948-016-9798-6

**Published:** 2016-06-27

**Authors:** Lisa Loikith, Robert Bauchwitz

**Affiliations:** Amerandus Research, 1735 Market Street, Suite 3750, Philadelphia, PA 19103 USA

**Keywords:** Research misconduct, Audit, ORI, HHS, Yellow Book, Inspector General, NSF, GAGAS, Conflicts of interest, Threats to independence

## Abstract

Nearly 90 % of allegations of biomedical research misconduct in the United States are dismissed by responsible institutions without any faculty assessment or auditable record. Recently, members of the U.S. Congress have complained that the penalties for those against whom findings of research misconduct are made are too light and that too few grant funds associated with research misconduct have been recovered for use by other researchers and taxpayers. Here we discuss the laws that empower federal agencies that can oversee investigations of biomedical research misconduct: the Office of Research Integrity (ORI) and the Office of the Inspector General (OIG), both located within the Department of Health and Human Services (HHS). Research misconduct investigations pertaining to U.S. physical sciences funded through the National Science Foundation (NSF) are overseen by the NSF’s OIG. While OIGs may provide some improvement over the ORI in the handling of research misconduct, we have found that a much more serious flaw exists which undermines an ability to conduct performance audits of the effectiveness by which allegations of research misconduct are handled in the United States. Specifically, sufficient data do not need to be retained by U.S. research institutions funded by HHS or NSF to allow effective audit of why allegations of research misconduct are dismissed before being seen by faculty inquiry or investigative committees. U.S. federal Generally Accepted Government Auditing Standards (GAGAS/Yellow Book), if applied to the research misconduct oversight process, would allow a determination of whether the handling of allegations of biomedical research misconduct actually functions adequately, and if not, how it might be improved. In particular, we propose that independent, external peer review under GAGAS audit standards should be instituted without delay in assessing the performance of ORI, or any other similarly tasked federal agency, in handling allegations of research misconduct.

**Electronic supplementary material:**

The online version of this article (doi:10.1007/s11948-016-9798-6) contains supplementary material, which is available to authorized users.

## Introduction

A recent article in the New York Times by two science journalists described the case of a researcher at Iowa State University who was arrested in June of 2014 and charged with four felony counts of making false statements (Marcus and Oransky 2014). The authors, who are the co-founders of Retraction Watch, noted that, “Even though research misconduct is far from rare”, such stringent outcomes are quite unusual for researchers in the U.S.: “most investigators who engage in wrongdoing, even serious wrongdoing, continue to conduct research at their institutions.”

Indeed, it was attention from the press and the U.S. senators from Iowa that probably led to the unlikely outcome for this researcher. As the authors noted, the accused researcher:

“may have remained one of the hundreds of fraudster scientists who faced little punishment if it weren’t for the attention of a senator. The three-year ban [proposed by the federal Office of Research Integrity, “ORI”], Senator Charles E. Grassley, Republican of Iowa, told [ORI] in a Feb. 10 [2014] letter, ‘*seems like a very light penalty* for a doctor who purposely tampered with a research trial and directly caused millions of taxpayer dollars to be wasted on fraudulent studies.’ (In fact, just two of the 11 cases reported by the O.R.I. last year led to outright bans. Most only required supervision by a scientist in good standing with research overseers.)”

[Italic font in quoted text within the pdf version of this article has been added for emphasis unless noted otherwise. Use of the definite article, “the”, before initialisms of agency names, such as ORI, will largely be dropped in keeping with HHS style guides, agency usage, and regulations referencing the agencies.]

The authors of the New York Times article then concluded with respect to ORI that: “The office needs teeth”, i.e. that ORI was in some way unable to impose harsher penalties or recover funds should it have desired to do so. Specifically, the authors proposed that “Congress should give [ORI] even more needed authority. A good starting point would be to grant the office the right to issue administrative subpoenas… Without subpoena power, the O.R.I. is able to see only what institutions want to share.”

We argue however, that the ORI is not significantly limited by law in the penalties it can already propose to the U.S. Secretary of Health and Human Services (HHS). Nor does ORI lack the means to obtain information from institutions, including those institutions that are non-compliant or not forthcoming.

Rather, it is more likely the orientation of the ORI towards remediation of scientists found to have engaged in misconduct, and perhaps most importantly, ORI’s conflicted role in support and education of the very institutions it is also supposed to regulate with respect to handling misconduct cases, that may have led to the unreasonably lax penalties like the one of which the senator recently complained. Indeed, present regulations appear to disallow ORI from conducting “direct” investigations, and ORI’s procedures have always made self-policing by research institutions the primary mechanism for fraud investigation.

Furthermore, rather than calling for more powers to be given to ORI, the U.S. senator most involved in the Iowa case called for the federal Department of Health and Human Services (HHS) to employ its Inspector General, who already has such law enforcement powers, in the fight against scientific fraud.

This article will first take a look at the laws affecting the powers of HHS’ ORI and its Office of the Inspector General (OIG) with respect to investigating scientific misconduct and imposing penalties. We then discuss how existing federal government audit standards might be applied to greatly improve the handling of allegations of research misconduct in the U.S. In particular, we note that for the over 87 % of biomedical research misconduct allegations which are dismissed without ever progressing to faculty inquiry or investigation, no specifically auditable evidence needs to be retained by responsible institutions or reported to the government.

## HHS Administrative Actions and Who May Impose Them

Under federal law 42 U.S.C. § 289b(c)(4), the Secretary of the U.S. Department of Health and Human Services is to establish regulations for actions to be followed by the Director of the ORI with respect to research misconduct.

The regulations authorized by 42 U.S.C. § 289b(c)(4) were published in the Federal Register (2005). Actions that can be taken by various HHS components, including potentially ORI, in response to research misconduct are defined in 42 CFR § 93.407:

- In response to a research misconduct proceeding [involving ORI; see § 93.402–406], HHS *may* impose HHS administrative actions that include but are *not limited to*:
  1. Clarification, correction, or retraction of the research record.
  2. Letters of reprimand.
  3. Imposition of special certification or assurance requirements to ensure compliance with applicable regulations or terms of PHS [the Public Health Service, a component of HHS that includes the NIH and FDA] grants, contracts, or cooperative agreements.
  4. Suspension or termination of a PHS grant, contract, or cooperative agreement.
  5. Restriction on specific activities or expenditures under an active PHS grant, contract, or cooperative agreement.
  6. Special review of all requests for PHS funding.
  7. Imposition of supervision requirements on a PHS grant, contract, or cooperative agreement.
  8. Certification of attribution or authenticity in all requests for support and reports to the PHS.
  9. No participation in any advisory capacity to the PHS.
  10. Adverse personnel action if the respondent is a Federal employee, in compliance with relevant Federal personnel policies and laws.
  11. Suspension or debarment under 45 CFR Part 76, 48 CFR Subparts 9.4 and 309.4, or both.
- In connection with findings of research misconduct, HHS also may seek to recover PHS funds spent in support of the activities that involved research misconduct.
- *Any authorized HHS component* may impose, administer, or enforce HHS administrative actions separately or in coordination with other HHS components, *including, but not limited to ORI*, the Office of Inspector General, the PHS funding component, and the debarring official.”

In addition, ORI can obtain research misconduct records from institutions under current law. Section 93.317 of 42 CFR Part 93 states that:

“(c) Provision for HHS custody. *On request, institutions must transfer custody of or provide copies to HHS, of any institutional record relevant to a research misconduct allegation covered by this part, including the research records and evidence,* to perform forensic or other analyses or as otherwise needed to conduct an HHS inquiry or investigation or for ORI to conduct its review or to present evidence in any proceeding under subparts D and E of this part.”

Thus, while the article by Marcus and Oransky (2014) argues that subpoena power would give ORI more of an ability to recover PHS funds or obtain documents from institutions, regardless of whether the National Institutes of Health (NIH) does or does not choose to act, they do technically already have that ability. However, there are other regulations which do appear to limit ORI to only recommending findings of misconduct and administrative penalties to the Office of the Assistant Secretary of Health (OASH).

## ORI’s Mandates

According to the following law from May 2000 which appears on the ORI website (as of November 27, 2015), ORI could no longer do its own “direct” investigations of research misconduct. Instead, it is supposed to either oversee those of the institutions it supports, or, for extramural grants (to such institutions), the HHS Office of the Inspector General (OIG) does the “direct” investigations (Federal Register 2000):

“… the *Assistant Secretary for Health (ASH)* will make proposed findings of research misconduct and administrative actions in response to allegations of research misconduct involving research conducted or supported by components of the Public Health Service (PHS);

*that direct investigations, previously conducted by ORI*, will be conducted by components of the PHS for intramural research and by *the Office of Inspector General for extramural research*;

and that the *role and structure of ORI will be changed to focus more on preventing misconduct and promoting research integrity through expanded education programs*.”

Furthermore, the Director of ORI only recommends to ASH whether to make findings of research misconduct and what administrative actions to “propose”. Presumably, this allows ASH to take into consideration other factors as to whether to act:

“E. Office of Research Integrity… The Director reports to the Secretary and will… (2) *recommend* to the Assistant Secretary for Health for decision, findings of research misconduct and administrative actions in connection with research conducted or supported by the PHS.” *Ibid*.

Those other factors might well include political and self-protective ones, as suggested by the February 25, 2014 resignation letter of David Wright, the Director of ORI since December 2011. Wright asked in his letter “whether OASH is the proper home for a regulatory office such as ORI, noting that [Assistant Secretary of Health/ASH] Koh himself has described his office as an ‘intensely political environment.’ (The contents of the letter were published in Kaiser 2014)

The law cited above is also notable for instructing ORI to “focus more” on education programs. The latter are run in association with the institutions that ORI is thought by the public to oversee with respect to research misconduct. The following is an excerpt from what Wright stated was the best part of his job:

“helping research institutions better handle allegations of research misconduct, provide in-service training for institutional Research Integrity Officers (RIOs), and develop programming to promote the Responsible Conduct of Research (RCR). Working with members of the research community, particularly RIOs… has been one of the great pleasures of my long career.” (Kaiser 2014).

Wright’s letter is instructive for seeming to miss the conflict of his desire (and mandate) to serve the research community by helping and supporting it, with the calls from others, including members of Congress, to provide sound anti-fraud oversight. Indeed, the ORI Handbook for Institutional Research Integrity Officers refers to its “partnership” “between itself and institutions” to handle scientific misconduct.

An important question, therefore, would be whether the mandate to serve as a consultant/educator hobbles ORI when it needs to get tough with large and powerful grantee-institutions. This sort of conflicted mandate to both serve and investigate/audit resembles the situation that existed for accounting firms which provided both consulting and auditing services to U.S. corporations before the Enron, Tyco, Adelphia, and WorldCom accounting scandals in the early part of this century led to the passage of the federal Sarbanes–Oxley Act of 2002 (SOX) which restricted such conflicted relationships.

Conflict of interest prior to SOX occurred because accounting firms were making relatively large amounts of money while acting as consultants; consequently such firms were reluctant to appear critical in audits of the same clients. Conflicts of interest, in general, are not restricted to acting with bias in favor of financial benefits relative to professional duties, nor is any actual improper action required. With respect to managing conflicts of interest by external audit firms, a significant component of SOX established standards for auditor independence. Categorizing and mitigating threats to independence, including conflicts of interest, are an important part of the 2011 revision of the federal Generally Accepted Government Auditing Standards (GAGAS), which are discussed in more detail below. (See “Audit Standards of Potential Relevance to the Improved Handling of Research Misconduct” section.)

Wright’s resignation as Director of ORI came just one day after he was to have responded to a letter from Senator Grassley that asked a number of questions about the function of ORI.

In a statement released to the press by Senator Grassley, he specifically cited the “inspector general” responsible for NIH, not ORI, as needing to become more involved in the investigation of research misconduct cases:

“The federal government has the authority to try to recover taxpayer dollars spent on research misconduct,” Grassley said. “Whether or not the government uses this authority and how much money it’s recovered in total are important questions. If this authority is under-used, the government could be wasting an opportunity to discourage the misuse of research dollars, in addition to not recovering what it should. The *inspector general* responsible for the National Institutes of Health *ought to be meaningfully engaged in misconduct cases*. With billions of research dollars at stake, we need to make sure all hands are on deck in preventing fraud and waste.” (Leys, see ESM.)

Senator Grassley’s specifically mentioning getting the HHS OIG, and not ORI, more involved in research misconduct investigations is consistent with a reading of the laws as presented above. [Agency usage of contiguous initialisms, such as HHS OIG, without indication of the possessive will generally be followed here unless emphasis is desired.]

Although the laws cited here suggest that it is HHS OIG that is to perform “direct” research misconduct investigations now instead of ORI, in practice, as suggested by the senator’s comments, HHS OIG does not typically conduct “direct” biomedical research investigations; (we have found at least one case in which they were peripherally involved: United States ex. rel. Dr. Helene Z. Hill v. University of Medicine & Dentistry of New Jersey, Dr. Roger W. Howell and Dr. Anupam Bishayee, 2:03-cv-04837-DMC). Rather, HHS OIG generally refers or defers cases to ORI with respect to biomedical research investigations, and ORI in turn relies upon the affected institution to investigate its own faculty, staff, or students. Why this might be is discussed in the following section.

Senator Grassley also appears to acknowledge that it was the political pressure of a “high profile” case, and probably attention from him and his senatorial colleague, that got action in this case:

“I started looking at the government’s response to federal research money lost to fraud after the Iowa State case. It was alarming to see weak oversight. The federal agencies that award these dollars haven’t been doing much to recover money lost to fraud in these cases or to hold anyone accountable. It’s encouraging to see an effort to increase oversight of taxpayer dollars. There should be more of this whenever federal research dollars are misspent, not just in a high profile case.”

In the following, a comparison is made of ORI and IG research investigations, after which federal audit standards that allow the involvement of true third-party, non-governmental reviews will be discussed.

## How do Research Misconduct Investigations by an Inspector General Compare to Those by ORI?

The preceding comments have addressed the way the U.S. federal law might impact the ability of ORI to address research misconduct (RM). As will be discussed below, a federal Inspector General’s office generally has law enforcement powers of the type that some have suggested be given to ORI.

However, it should also be noted that having power is one thing, while having motivation to use it is another. The reality of how HHS OIG uses its biomedical research fraud investigation and law enforcement powers, as opposed to deferring to ORI and thereby in large measure to the institutions supposedly being overseen by ORI, might suggest that Senator Grassley really was correct that *all* hands should get on deck.

We have posed several questions directly to HHS OIG including: (1) Does HHS OIG have a list of extramural biomedical research fraud cases that it has handled? If so, where is the list? Can we obtain it? (2) If such cases have been taken, under what criteria does HHS OIG make a direct investigation? (3) How are research misconduct allegations audited by the federal government? The reply received was:

“Please visit oig.hhs.gov to review all public information about OIG’s work.

Our *Work Plan* may be of particular interest; it is available at http://oig.hhs.gov/reports-and-publications/workplan/index.asp. (A new edition of the *Work Plan* will be posted in a couple of weeks.)

Thank you for your interest in OIG’s work.

OIG Public Affairs”

As no relevant information was found on the HHS OIG website, we instead examined the performance of the National Science Foundation’s OIG, which acts against research misconduct in the absence of an ORI-like analog.

For most of this comparison, we reference a talk by the NSF OIG’s Dr. Jim Kroll, Director of the Research Integrity and Administrative Investigations Unit (Kroll 2014), as well as answers to questions he provided directly to us (October 20, 2014).

First, Dr. Kroll noted in his talk with respect to his own program that “OIG is delegated the responsibility for investigating research misconduct allegations involving NSF programs”.

Unlike the highly politicized environment of HHS OASH about which the former ORI Director complained, NSF OIG reports directly to Congress and the National Science Board (NSB), the latter being made up of eminent scientists who have an advisory role.

Kroll also noted what he calls “some subtle differences” between ORI and NSF OIG:

1. NSF has the “Ability to independently investigate” whereas ORI “Oversees grantee investigations”. This supports our review of the law, under which, as amended, ORI does not do direct investigations.
2. NSF OIG is a law enforcement (LE) agency “with subpoena authority [and] Search warrant capability (criminal)” while ORI is not an LE agency. This relates to some of the power that Marcus and Oransky (2014) had proposed for ORI.
3. NSF OIG has “Limited outreach by investigative staff” while ORI has a “Division of Education/Integrity” - which as previously noted we consider a major structural flaw in need of a strong SOX-like separation of consulting and audit operations.

NSF OIG’s Kroll did not have knowledge of any significant investigation or audit of biomedical research fraud by HHS OIG.

### Research Misconduct and Fraud

As to the question of how research misconduct compares to fraud, Kroll defined fraud as “A misrepresentation of material fact to induce another to act to their detriment.” NSF OIG determines intent essentially by scienter standards: “Must be with a culpable intent (reckless, knowing or purposeful, not careless)”.

If one restricts research misconduct to fabrication and falsification (F&F), it is fairly straightforward to see how those who are misled by material F&F would be defrauded by it, i.e. by relying upon such false information to take any act, such as new experiments, and not merely by the act of agreeing to fund the fraudsters’ research.

Thus, it can be surmised that the consequences of research misconduct and fraud are often one and the same. Consequently, Dr. Kroll lists the NSF OIG responses to research misconduct as follows: “If NSF awards funds based on a proposal containing research misconduct—the case is analyzed under the criminal and civil fraud statutes and common law fraud doctrine.” Some of the statutes that the NSF OIG considers relevant to research misconduct include:

Conspiracy 18 U.S.C. §371

False Claims 18 U.S.C. §287

Embezzlement 18 U.S.C. §641

Theft of Federal Funds–18 U.S.C. §666

False Statements 18 U.S.C. §1001

Mail Fraud –18 U.S.C. §1341

Wire Fraud 18 U.S.C. §1343

Civil False Claims –31 U.S.C. §3729(a)

However, as noted above, it appears that ORI has been given a primary mandate to cooperate with and support research institutions. Thus, a stringent fraud investigation posture by ORI might be taken by some biomedical research institutions as antithetical to their interests. Kroll appears to address this potential conflict of interest by noting that it should not be considered problematic for each party to pursue its perceived mandates and objectives; ultimately, any conflicts should be adjudicated: “Adjudication—*Institution should act only to protects [sic] its interests; OIG makes recommendations to protect federal interests*”. The latter statement is taken here to mean that NSF OIG’s interests with respect to research misconduct are not necessarily aligned with the university’s—or at least not with short-term university interests, as some who run a university might see it in financial and reputational terms.

With respect to resolving such conflicts of interest should they arise, it is the NSF and its Director who do the adjudication. This might seem to give uncooperative or antagonistic research institutions an edge over OIG because of connections and access that the regulated institutions might ordinarily have with such adjudicating officials. Indeed, in the recently resolved Feldheim Eaton case, the NSF OIG’s finding of research misconduct was reversed by the NSF Director’s Office. We provide additional analysis as to why this might have occurred elsewhere (Bauchwitz 2016).

Although such an adjudication mechanism might not be considered an ideal solution, nevertheless, it should still be worthwhile to compare NSF OIG’s track record in rooting out research fraud with ORI’s.

Comparing research misconduct investigations by NSF OIG and ORI, we find a dramatic difference. For NSF, case statistics show that from 2003 through 2010 there were almost twice as many research misconduct F&F findings against PI (principal investigator) and co-PIs than postdoctoral fellows and students (mean 7.0 PI/co-PI; 3.25 PD/student; 2-tail *t* test *p* = 0.002; data was taken from the Kroll 2014). This would be the opposite of the case for ORI, for which probit analysis of the data showed that there was a statistically significant increase in the likelihood of biomedical research misconduct findings against postdoctoral fellows, graduate students, and staff compared to professors of all ranks (Pozzi and David 2007).

The apparent less frequent findings of research misconduct against higher level faculty in the biomedical sciences compared to the physical sciences might reflect the superior oversight provided by NSF OIG, or for some reason a greater reticence that biomedical research institutions could have in taking meaningful action against PIs whose demise could produce negative reputational and financial consequences to the institutions. However, there may be deeper flaws in the handling of research misconduct allegations in the U.S., regardless of whether an OIG is providing oversight.

Even if HHS OIG were pushed to become more active in investigating research misconduct cases, it might be asked whether HHS OIG would prove to be significantly more resistant to political pressures than ORI has apparently been. At least OIG’s mandate is probably more consistent with those of professional fraud investigators than the conflicted one ORI now has, e.g. OIG’s follow federal audit and IG standards (see below). Furthermore, a key mechanism federal OIGs employ to reduce the threat to the independence of their investigation is that “IGs report to both the head of their respective agencies and to the Congress”… It is the ability for independent oversight by Congress that is believed to be a “legislative safety net that protects the IG’s independence and objectivity.” (CIGIE 2012).

Inspectors General, however, have also been subject to pressure before and have had a history consistent with a lack of complete independence from their departments (Light 1993). The fact that HHS OIG is deferring to ORI and OASH in the handling of biomedical research misconduct investigations supports the view that they are in a cooperative position with their department, regardless of what the law may state or what adequate audit of department performance (in this case ORI) may warrant.

Even more worrisome is that administrative pressures against many federal OIG’s in recent years may have reached a new crisis point:

“[A] formal opinion in July from the [Department of Justice’s] Office of Legal Counsel… which applies to federal agencies across the government, concluded that the 1978 [OIG] law giving an inspector general access to ‘*all records*’ in investigations did not necessarily mean *all records* when it came to material like wiretap intercepts and grand jury reports… ‘The bottom line is that we’re no longer independent,’ Michael E. Horowitz, the Justice Department inspector general, said in an interview… The administration insists there is no intention of curtailing investigations, but both Democrats and Republicans in Congress have expressed alarm and are promising to restore full access to the watchdogs.” (Lichtblau 2015).

Thus, it is possible, if not likely, that additional, more U.S. administration-independent anti-fraud mechanisms would be useful in order to increase the likelihood that fraud against taxpayers is exposed and lost grant funds recovered. One such avenue already exists, and perhaps not coincidentally, it was Senator Grassley who was involved with its reinvigoration in 1986: The federal False Claims Act (FCA; 31 USC 3729-3733; further amended by the Fraud Enforcement and Recovery Act of 2009 and Patient Protection and Affordable Care Act of 2010). However, even the use of the FCA in handling research misconduct has proved very difficult given the small percentage of such cases actively pursued by the U.S. Department of Justice (DOJ) and the undue influence that ORI can have on such cases while acting as “investigators” for the Department of Justice.

The primary intent of the federal False Claims Act is to allow recovery of taxpayer funds from anyone who “knowingly presents, or causes to be presented, to an officer or employee of the United States Government or a member of the Armed Forces of the United States a false or fraudulent claim for payment or approval.” This quite clearly covers the presentation of grant applications containing knowingly false information to the U.S. National Institutes of Health.

The Attorney General of the United States, who is the head of the Department of Justice (DOJ), is tasked with pursuing cases under the FCA. With respect to how DOJ can choose others to assist it in investigations, the FCA states that the AG can use “designees” to assist with investigative activities (specifically citing the issuance of civil investigative demands, which are akin to subpoenas). However, there is not any limitation in the FCA on whom the DOJ can appoint as its investigative designees, including those, such as ORI, who may have already failed to act or otherwise have potential conflicts of interest (as discussed above).

Next, the biomedical research misconduct process is briefly discussed to provide a framework of its various stages.

## The Biomedical Research Misconduct Legal Definition and Process

Research misconduct is defined by U.S. federal law 42 CFR Part 93 **§**103 as “fabrication, falsification, or plagiarism in proposing, performing, or reviewing research, or in reporting research results.

- Fabrication is making up data or results and recording or reporting them.
- Falsification is manipulating research materials, equipment, or processes, or changing or omitting data or results such that the research is not accurately represented in the research record.
- Plagiarism is the appropriation of another person’s ideas, processes, results, or words without giving appropriate credit.”

The first step in handling biomedical research misconduct allegations made to U.S. academic institutions is typically an assessment by the institution’s designated Research Integrity Officer (RIO) (ORI 2011a). The RIO works in conjunction with a Deciding Official (DO) (ORI 1997), who can be a dean. For example, an assessment procedure published by a large U.S. academic biomedical research institution has stated:

“V. PROCEDURES: CONDUCTING THE ASSESSMENT AND INQUIRY

A. Allegations. Any report of alleged or apparent research misconduct should be brought *immediately* to the attention of the RIO who will promptly, in consultation with the DO, *assess the allegation* to determine whether it is sufficiently credible and specific so that *potential evidence of research misconduct may be identified* and whether the allegation falls within the definition of research misconduct in this policy. *An inquiry must be conducted if these criteria are met*. In the event that the RIO and the DO disagree as to whether the inquiry should be conducted, an inquiry will be conducted.” (Weill Cornell 2007).

The preceding process conforms to the requirements for an inquiry under federal law (42 CFR Part 93 §307), i.e. that an inquiry is warranted if the allegation:

1. Falls within the definition of research misconduct under this part;
2. Is within § 93.102 [in brief, it applies to Public Health Service supported research, training, and proposals made]; and
3. Is sufficiently credible and specific so that potential evidence of research misconduct may be identified.”

The biomedical research misconduct inquiry has a relatively low standard of proof, as the NSF OIG stated is also the case for inquiries handled under its purview. A more detailed explanation of the purpose of a research misconduct inquiry is provided from the same university procedures cited just above:

“Initiation and Purpose of the Inquiry. If the RIO determines that the criteria for an inquiry are met, he or she shall promptly initiate the inquiry process. The purpose of the inquiry is to conduct an initial review of the available evidence to determine whether to conduct an investigation. An inquiry does not require a full review of all the evidence related to the allegation. An investigation is warranted if there is a reasonable basis for concluding the allegation falls within the definition of research misconduct and the preliminary information gathering and fact finding from the inquiry indicates that the allegation may have substance.” (Weill Cornell 2007).

In practical terms, what is important about an inquiry is that it is the first point at which the faculty more broadly and officially are made aware of and become involved in the handling of allegations, and the first point at which specific records of the allegations must be retained. It seems evident that the initial assessment is primarily intended to eliminate unquestionably frivolous allegations or those that are not related to fabrication, falsification, or plagiarism.

## The Percentage of Dismissed Allegations of Biomedical Research Misconduct is Remarkably High

Biomedical faculty, students, and staff have been receiving training on the definition of, and how to handle, research misconduct beginning prior to the establishment of ORI in 1992 (ORI 2011b). Consequently, it would not be expected that a high percentage of unsubstantiable allegations would be made, particularly given the very severe consequences that may accrue to those making such allegations, even when meritorious (e.g. see Wilmhurst 2007; Martin 2013; Sullivan 2012; McMillan 2012; O’Rourke 2012; Rothschild and Miethe 1999).

Remarkably, however, we found from an examination of ORI data on the handling of research misconduct that almost 90 % of biomedical research misconduct allegations continue to be dismissed without receiving an initial inquiry or generating any other specific record or detailed report to ORI. Of 3561 allegations of research misconduct made between 1994 and 2011 in the United States, only 475 (13.3 %) received any form of inquiry or investigation, including those administratively closed, for which ORI may have concluded that no more evidence would have been found (Table 1). Only 12.6 % of allegations of biomedical research misconduct went before a faculty inquiry or investigative committee; 87.4 % were dismissed as frivolous, not involving PHS funding, or not even potentially meeting the standards of research misconduct. (Data were taken from the ORI’s Annual Reports which present outcomes of research misconduct cases for U.S. institutions.)

**Table 1:** Handling of U.S. biomedical research misconduct allegations 1994–2011

Year case closed	Total allegations	Inquiry, no investigation	Investigation, no research misconduct	Investigation, research misconduct	Inquiry, administrative closure*	Investigation, administrative closure*	Total closed
2011	240	1	15	13			29
2010	155	1	20	9		1	31
2009	179	1	31	11			43
2008	201		4	13			17
2007	217	5	8	10		5	28
2006	266	5	9	15	2	4	35
2005	265	3	10	8	1		22
2004	267	7	8	8			23
2003	179	5	12	12			29
2002	191	7	9	13	2	1	32
2001	196	6	4	14		1	25
2000	173	12	6	7	1	1	27
1999	129						33
1998	112						32
1997	166		14	14		1	29
1996	196		19	17		2	38
1995	244		14	24		3	41
1994	185		14	11		1	26
Total	3561	53	197	199	6	20	540

The data presented in Table 1 are consistent with those presented by Pozzi and David (2007), who proposed that “The numbers suggest that the evidence required to open an inquiry must be quite strong.” As noted above, however, the intent of the initial assessment, and even a faculty inquiry, is not meant to set a high bar to inquiry or investigation; otherwise, as few as two individuals in an institution (typically the RIO and DO, often a dean) can prevent general faculty examination of allegations.

Unfortunately, there is no way at present for the public, or even for an IG such as that of the NSF, to determine why allegations of NIH- or NSF-funded research misconduct were dismissed by U.S. research institutions. A review of U.S. law suggests how this situation arose for ORI.

## Only Aggregate Data on Allegations of Biomedical Research Misconduct Need be Retained

In its 1989 report on research misconduct (“Misconduct in Scientific Research”**)**, HHS OIG recommended with respect to allegations that:

“regulations issued by the Department should *require* that grantee institutions *immediately notify* the Department whenever they detect or receive an allegation of scientific misconduct” (HHS OIG 1989).

U.S. federal law appears to be consistent with the HHS OIG recommendation quoted above:

“Section 493 of the Public Health Service Act, as amended by Pub. L. 99–158, the Health Research Extension Act of 1985, provides that the Secretary by regulation *shall require* that each entity that applies for a grant, contract or cooperative agreement which involves the conduct of biomedical or behavioral research shall submit *an approved assurance*. This assurance developed under the regulation promulgated to implement Pub. L. 99–158 states that the institution 1) has established policies and procedures to review, investigate and *report allegations* of research misconduct in connection with biomedical and behavioral research conducted at or sponsored by the applicant institution with PHS supported funds, 2) will comply with its own policies and *will report to the Secretary* any investigation or *alleged misconduct* and 3) will follow the requirements of 42 CFR Part 50, Subpart A which has been superseded by 42 CFR Part 93.”

Nevertheless, the actual allegation information provided to ORI appears to be in aggregate form only:

“To keep its assurance active, each institution must submit to ORI an Annual Report on Possible Research Misconduct (PHS Form 6349) that provides *aggregate information* *on allegations*, inquiries, investigations, and other activities required by the PHS regulation.” (ORI Annual Reports 2011).

Federal law 42 CFR 93.309(c) specifies with respect to “Documentation of decision not to investigate” that “Institutions must keep sufficiently detailed documentation of *inquiries* to permit a later assessment by ORI of the reasons why the institution decided not to conduct an investigation”.

Similarly, 42 CFR 93.317, “Retention and custody of the research misconduct proceeding record”, only specifies retention of records associated with institutional inquiries and investigations. ORI’s “Sample Policies and Procedures for Responding to Allegations of Research Misconduct” does not specify any procedure for documentation of allegations (ORI 2012).

Surprisingly, this lack of requirement to retain allegation records is not merely a failure specific to the U.S. biomedical research establishment. When the NSF IG was asked if it would be possible to produce an audit of allegations made to the research institutions it oversees, the response was that institutions and private businesses do not have to report all allegations to NSF OIG. Institutions can perform an inquiry, which, as noted above, NSF OIG’s Kroll stated should have a low standard to meet. If, however, there is no substance to the allegation, it does not have to be passed on to NSF OIG. Most importantly, NSF OIG stated that they have no idea, and believes no one is auditing, how allegations among its grantees are handled prior to the inquiry stage. (Conversation of October 20, 2014, *ibid*.)

Therefore, a specific record is not required if an academic institution overseen by ORI or NSF dismisses an allegation of research misconduct prior to an inquiry. As a result, close to 90 % of U.S. biomedical research allegations are dismissed and almost completely left without a record. Thus, most allegations of research misconduct made in the U.S. are highly unlikely to be auditable with respect to whether they were properly handled.

Reviewers should not be left in a position to have to guess at the reasons for the dismissal of allegations of research misconduct. There are federal standards for auditing, presented below, which would allow review of the handling of allegations of research misconduct.

## Audit Standards of Potential Relevance to the Improved Handling of Research Misconduct

In the following, we examine U.S. federal government audit standards that might pertain or be applied to assessment of the performance of the institutional research misconduct process and ORI’s (or an OIG’s) oversight of that process. Of primary note are the Government Accountability Office’s (GAO’s) “generally accepted government auditing standards” or “GAGAS” (GAO 2011). These standards, also referred to as the Yellow Book, underlie the more investigation-specific standards found in the government’s “Quality Standards for Federal Offices of Inspector General”, known as the Silver Book (CIGIE 2012).

The federal government’s audit standards provide some important direction on issues pertaining to assessing and managing potential conflicts of interest which might affect regulatory bodies, such as those discussed above for ORI. For example, GAGAS states:

“Maintaining objectivity includes a continuing assessment of relationships with audited entities and other stakeholders in the context of the auditors’ responsibility to the public. The concepts of objectivity and independence are closely related. *Independence impairments impact objectivity*.”

Of particular importance with respect to ORI and its dual roles of institutional support and oversight, it is not sufficient to simply declare there is independence because functions such as support and investigation are in separate “divisions”, as is currently the case for ORI:

3.10 “for the purposes of independence evaluation using the conceptual framework, an audit organization that includes multiple offices or units, or includes multiple entities related or affiliated through common control, is considered to be one audit organization.”

More specifically, GAGAS provides additional guidance as to actions that true auditors should take to address threats to their independence:

“3.14 Threats to independence may be created by a wide range of relationships and circumstances. Auditors should evaluate the following broad categories of threats to independence when threats are being identified and evaluated:

a. Self-interest threat - the threat that a financial or other interest will inappropriately influence an auditor’s judgment or behavior;

b. Self-review threat - the threat that *an auditor or audit organization that has provided nonaudit services* will not appropriately evaluate the results of previous judgments made or services performed as part of the nonaudit services when forming a judgment significant to an audit;

c. Bias threat - the threat that an auditor will, as a result of political, ideological, social, or other convictions, take a position that is not objective;

d. Familiarity threat - the threat that aspects of a relationship with management or personnel of an audited entity, such as a close or long relationship, or that of an immediate or close family member, will lead an auditor to take a position that is not objective;

e. Undue influence threat - the threat that external influences or pressures will impact an auditor’s ability to make independent and objective judgments;

f. Management participation threat - the threat that results from an auditor’s taking on the role of management or otherwise performing management functions on behalf of the entity undergoing an audit; and

g. Structural threat - the threat that an audit organization’s placement within a government entity, in combination with the structure of the government entity being audited, will impact the audit organization’s ability to perform work and report results objectively.”

Self-review threat could encompass the conflict of interest arising from the services and duties of ORI’s Division of Education and Integrity and its Division of Investigative Oversight (DIO). For example, if ORI’s education and support division believes that it has established that an institution is doing a good job of training and taking other steps against research misconduct, then there might be a bias against the investigative division making findings that suggest otherwise. We previously compared this situation to the one that led to passage of the Sarbanes–Oxley Act, which restricted audit firms from providing non-audit services to the same client.

Structural threat was suggested when former ORI Director Wright questioned whether the placement of ORI in the highly politicized environment of OASH at HHS was appropriate. (Kaiser 2014).

Also of note, self-interest threats are raised by the potential conflict that can arise when an institution and its deans or other officials have professional or financial interests in the grants being obtained by faculty who have been accused of research misconduct. The NSF case referenced above (Bauchwitz 2016) involved evidence of a similarly conflicted process. In that case, the NSF OIG criticized the investigation performed by faculty investigative committees at two universities concerning a faculty member who had been hired by one of the universities (the University of Colorado, Boulder) from the other (North Carolina State University). From an article on the NSF OIG report by a journalist:

“The [NSF] inspector general’s report also pointed to lapses in the internal investigations. The N.C. State investigative committee, for example, did not examine [graduate student] Gugliotti’s lab notebooks, which were the basis of the Science article.

The University of Colorado investigation had more problems, according to the report. For one, it inaccurately said that N.C. State had “exonerated” Feldheim and Eaton. N.C. State had found that Feldheim acted negligently and that all three published false statements.

The inspector general said Colorado failed to contact key witnesses and accepted the promises of Feldheim and Eaton to correct the record, which they have not done.

Joseph Rosse, head of research integrity and compliance at Colorado, declined to be interviewed.” (Neff 2016).

The unwillingness of a research integrity officer to respond to a well-known journalist who covers scientific misconduct matters illustrates the benefits of using professional auditors such as those in an OIG who can require responsiveness under law.

This NSF OIG case even more starkly shows just how inappropriate the results of such conflicts of interest can become. One of the UC Boulder investigative committee members apparently made derogatory comments to the press about a professor who had originally acted as a whistleblower in bringing the research misconduct concerns to light:

“The investigation was led by Colorado physics professor Alan Franklin, who told The Daily Camera of Boulder in 2014 that [NC State Professor Stefan] Franzen had ‘made a career’ of complaining about Feldheim and Eaton.

‘There’s been great damage done to people, mainly Eaton and Feldheim, for no good reason,’ Franklin told the newspaper.

Contacted this week, Franklin declined to discuss the final NSF reprimand, saying it was a confidential personnel matter.

Franzen welcomed the NSF ruling as vindication of his struggle to correct the record but said he remains bitter at how he says Feldheim, Eaton and their allies have sullied his reputation for years. Instead of addressing the underlying science, he said, his adversaries hired lawyers to gum up and prolong the investigations and resorted to personal insults to slander him.” (Neff 2016).

Even the NSF OIG is apparently willing to risk self-review threat by returning the handling of allegations of research misconduct back to an affected university:

“Whenever possible, OIG also relies on the relevant professional community to evaluate the seriousness of alleged misconduct based on its accepted standards and practices. To achieve this, we often refer investigations to the institution managing the award for evaluation.”

It is not clear that allegations referred back to an institution by NSF OIG would necessarily proceed to the level of faculty inquiry.

Institutional self-investigation also falls into what is more commonly referred to as “self-policing” or, more formally, the conflict of interest known as group or industrial self-regulation. With respect to actual law, most of those at the federal level deal with financial conflicts of interest of individual government employees. (Maskell 2014). There is limited law that addresses organizational conflicts of interest (COI), such as 48 CFR subpart 9.5, federal “Organizational and Consultant Conflicts of Interest” law, which is cited by the Centers for Medicare and Medicaid Services (CMS) and the Department of Defense. While this law might be used to address conflicts of interest from a research institution providing grant services and also investigating misconduct associated with those services, it does not deal with potential intra-agency or inter-agency COI that could be relevant to the structure of ORI. For this reason, we believe that the standards set by GAGAS against threats to independence should be codified into federal research misconduct oversight laws.

Ultimately, we recommend that university faculty should no more be relied upon to investigate their colleagues than they would be allowed to vote on their federal grant funding. At a minimum, HHS OIG could convene investigative review committees, just as NIH runs grant review committees. Alternatively, or in combination, independent, investigative and audit firms could be employed, as in the SOX model.

There are a number of federal audit guidelines for addressing the aforementioned threats to independence and conflict of interest. Some of those involve the important concept of performance audits:

“2.10 Performance audits are defined as audits that provide findings or conclusions based on an evaluation of sufficient, appropriate evidence against criteria.”

Performance audits are also more broadly of value in determining whether an oversight system is actually functioning adequately.

One example of a performance audit involving the handling of research misconduct that can be obtained by online search was produced by the Department of Energy’s OIG in 2014 (DOE 2014). That audit, however, stated that it “did not review the actual allegations”. As part of this study, DOE’s OIG was asked whether they would “have been able to review all allegations of research misconduct within its purview had it chosen to do so”, i.e. “would sufficient documentation per GAGAS 6.56 and 6.57 be retained by responsible entities in order for DOE OIG to assess all allegations, including in particular those dismissed prior to any inquiry”. However, DOE’s OIG declined to answer, writing that, “The posed questions exceed the scope of our audit”. They also did not specify, in response to a question, any law that would have required such documentation of allegations. (Written response to questions from F. Jones, DOE OIG, April 29, 2016).

An example of a performance audit that included delving into actual allegations was produced by the Colorado Office of the State Auditor (Colorado OSA 2016). In this case, the allegations were ethics complaints made against state judges, but the concept and process would apply equally to scientists. Interestingly, legislation to create a federal OIG for the U.S. judiciary has now been introduced into Congress (S. 1418 2015). Thus, the proposals made in this article are part of a more generalized approach to improve government function using IGs and audits.

Audits do not need to be conducted by governmental bodies, but can involve third party reviews in order to independently confirm performance. GAGAS discusses what it terms “safeguards, which are used to mitigate threats to auditor/investigator independence”; these include reviews by external entities:

“3.16 Safeguards are controls designed to eliminate or reduce to an acceptable level threats to independence.

3.17 Examples of safeguards include:

- consulting an independent third party, such as a professional organization, a professional regulatory body, or another auditor;
- involving another audit organization to perform or reperform part of the audit.”

The preceding “examples”, while potentially very sensible, are not obviously mandated. However, there is some required quality control and assurance in GAGAS 3.82. Of note:

“Each audit organization performing audits in accordance with GAGAS must:

b. have an external peer review performed by reviewers independent of the audit organization being reviewed *at least once every 3* *years*.”

Characteristics of an external peer review are specified in GAGAS 3.105:

“An external audit organization should make its *most recent peer review report publicly available*. For example, *an audit organization may satisfy this requirement by posting the peer review report on a publicly available web site* or to a publicly available file designed for public transparency of peer review results. Alternatively, if neither of these options is available to the audit organization, then it should use the same transparency mechanism it uses to make other information public. The audit organization should provide the peer review report to others upon request.”

Such peer reviews of government IG audit are sometimes published as a short letter, and frequently are performed by another federal audit IG. What we propose here is a much more detailed, non-governmental third party review that completely conforms to GAGAS and is published, to the extent possible (see next), in its entirety.

A very common objection made against the publication of raw audit information is that it will undermine confidentiality. GAGAS also addresses this issue:

“5.40 Certain information may be classified or may be otherwise prohibited from general disclosure by federal, state, or local laws or regulations. In such circumstances, *auditors may issue a separate classified or limited use report* containing such information and distribute the report only to persons authorized by law or regulation to receive it.”

Therefore, at a minimum, independent auditors should see all confidential information, even if laws prevent the public from seeing the same. External peer review auditors may have to publish such details in a restricted manner. Thus, ORI, or any other oversight entity such as HHS OIG, should be provided with confidential information regarding allegations when auditing the performance of institutions and ORI in handling them, and the same information should be made available to independent third party reviewers who engage in performance audits of ORI and its handling of biomedical research misconduct allegations.

A related and very major issue is the retention of adequate data for performance audits:

“6.01 This chapter contains field work requirements and guidance for performance audits conducted in accordance with generally accepted government auditing standards (GAGAS). The purpose of *field work requirements* is to establish an overall approach for auditors to apply in obtaining reasonable assurance that the *evidence is sufficient and appropriate* to support the auditors’ findings and conclusions.”

As noted previously, we strongly question whether the use of aggregate data alone to assess allegations of biomedical research misconduct could provide sufficient evidence for any sort of audit. More definitively, GAGAS contains the following requirement:

“6.56 Auditors *must* obtain sufficient, appropriate evidence to provide a reasonable basis for their findings and conclusions.

6.57 The concept of sufficient, appropriate evidence is integral to an audit. Appropriateness is the measure of the quality of evidence that encompasses its relevance, validity, and reliability in providing support for findings and conclusions related to the audit objectives. In assessing the overall appropriateness of evidence, auditors should assess whether the evidence is relevant, valid, and reliable. Sufficiency is a measure of the quantity of evidence used to support the findings and conclusions related to the audit objectives. In assessing the sufficiency of evidence, auditors should determine whether enough evidence has been obtained to persuade a knowledgeable person that the findings are reasonable.”

If such a standard were specified in U.S. federal research misconduct laws, we believe that the resulting evidence could permit a valid assessment of current procedures to handle allegations of research misconduct.

## Conclusion

There have been recent calls from Congress for the Department of Health and Human Services to recover more funds and provide stronger penalties in biomedical research misconduct cases. Others have suggested that HHS’s Office of Research Integrity be given additional powers in order to accomplish such objectives. As reviewed here, ORI is limited by law in its ability to directly investigate misconduct cases. We also note that ORI could be at risk from a SOX-type conflict of interest between its educational-consulting-support and investigative oversight operations; this too could further limit its willingness to pursue cases. It has been proposed by a U.S. Senator who was involved with the 1986 revitalization of the federal False Claims Act that the HHS’ Office of Inspector General become more involved in biomedical research misconduct cases since it already has law enforcement powers of the type proposed for the ORI.

We believe that one means to resolve several of these concerns would be to move the ORI’s Division of Investigative Oversight into the HHS’s OIG. In this way, federal biomedical research misconduct investigators/overseers would be required to follow federal IG and audit standards, have enhanced law enforcement powers, have a reduced potential conflict of interest with ORI’s educational division, and also have access to Congress, all as now exist for the National Science Foundation’s OIG in its oversight of research misconduct in the physical sciences.

In general, we believe that the GAGAS standards on threats to independence mark a significant step forward in addressing conflicts of interest involving governmental agencies and the entities they oversee. Such standards should be codified into federal research misconduct (and other relevant) laws, and not be limited to professional audit entities which follow GAGAS. One consequence of doing so might be to move away from institutional research misconduct self-investigations.

Importantly, regardless of whether HHS’s OIG is used or ORI’s DIO is retained, performance audits, including by independent third parties, would be of critical value in determining whether an oversight system is actually functioning adequately. At present, however, sufficient data do not appear to be retained to allow effective audit of the handling of most allegations of research misconduct in the U.S. As noted in GAGAS, auditors, like scientists, “must obtain sufficient, appropriate evidence”. Consequently, we believe that there is a pressing need - including in the physical sciences - to retain all evidence related to allegations made of research misconduct and how those allegations were handled prior to a decision to dismiss or pass them on to faculty inquiry. With such evidence, we believe that U.S. federal audit standards exist which would permit appropriate assessment of the handling of research misconduct investigations.

## Electronic supplementary material

Below is the link to the electronic supplementary material.

Supplementary material 1 (pdf 319 KB)
