# Supplementary material for: The Essential Need for Research Misconduct Allegation Audits
Source: Sci Eng Ethics. 2016 Jun 27;22(4):1027–49. doi: 10.1007/s11948-016-9798-6 (PMC4996876; doi:10.1007/s11948-016-9798-6)
Supplement: Supplementary file 1 — Supplementary material 1 (pdf 319 KB) [file 11948_2016_9798_MOESM1_ESM.pdf]

ADVERTISEMENT

Ethics expert: ISU researcher should be prosecuted

Jan. 18, 2014 | 7 Comments

Recommend Be the first of your friends to recommend this.

Recommend 0 Tweet 2 +1 0 A A

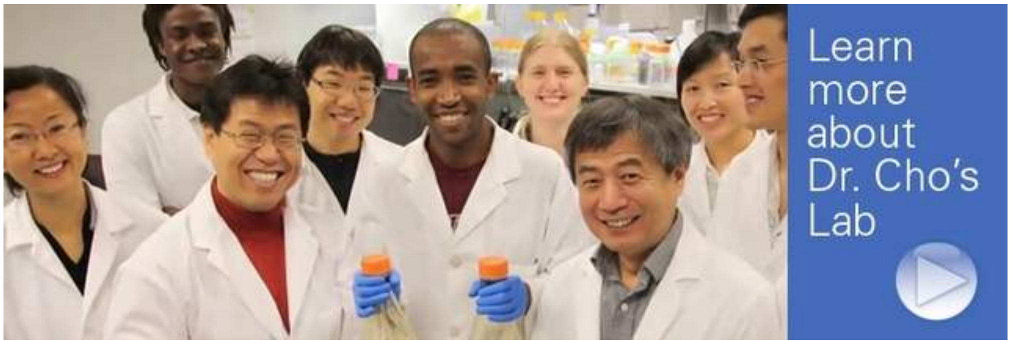

Dong Pyou Han, front right, stands with the rest of an ISU research team led by Michael Cho, front left, in a picture promoting their work to develop an AIDS vaccine. Han resigned last fall after admitting faking results of experiments. / Special to the Register

ADVERTISEMENT

Written by **Tony Leys**

FILED UNDER

News

Someone should have notified law enforcement authorities that an Iowa State University scientist helped bring in millions of federal grant dollars by blatantly faking AIDS-vaccine results, a national research-ethics expert said Friday.

“Fraud like this, involving significant sums of taxpayer money and harm to people who rely on this information ... should be criminally prosecuted,” said Arthur Caplan, a bioethics professor at New York University.

The scientist, Dong Pyou Han, resigned from ISU last fall. He admitted that he spiked rabbit blood with human antibodies to make it appear that a vaccine was protecting the animals against HIV, the virus that causes AIDS. He also admitted falsifying data in reports.

This was not a run-of-the-mill case of plagiarism or a researcher fudging a few numbers in a paper, Caplan said. “Here,

Harkin and Grassley weigh in on fraud case

U.S. Sen. Tom Harkin, an Iowa Democrat who helps oversee the National Institutes of Health, expressed disappointment Friday about the discovery of research fraud at Iowa State University. “I fight hard for this funding, so it is particularly upsetting for me to hear allegations of it being misused. This case should be

thoroughly examined and all appropriate measures taken to safeguard taxpayer funds and ensure that funds are not diverted from truly lifesaving research," he said in a prepared statement.

Iowa's other U.S. senator, Republican Chuck Grassley, also released a statement in response to a Register query about the case: "The federal government has the authority to try to recover taxpayer dollars spent on research misconduct," Grassley said. "Whether or not the government uses this authority and how much money it's recovered in total are important questions. If this authority is under-used, the government could be wasting an opportunity to discourage the misuse of research dollars, in addition to not recovering what it should. The inspector general responsible for the National Institutes of Health ought to be meaningfully engaged in misconduct cases. With billions of research dollars at stake, we need to make sure all hands are on deck in preventing fraud and waste."

Spokeswomen for both senators said they were unsure if law-enforcement authorities should have been brought into the matter.

Less

ADVERTISEMENT

you've got just flat-out fraud, like somebody doctoring a credit card."

University documents released to The Des Moines Register this week suggest that Han, 56, likely returned to his native Korea after quitting. If so, Caplan said, it could be tough for authorities to prosecute Han even if they wanted to.

The documents Iowa State released this week include scores of emails between ISU officials and federal research administrators. The emails were exchanged last year after outside scientists questioned the results of the ISU vaccine research. ISU and federal officials discussed at length how to determine who was at fault and then what administrative steps to take against Han. The emails don't include discussion of reporting the matter to law enforcement.

University spokesman John McCarroll said he did not believe ISU alerted criminal prosecutors, or that it should have been expected to. McCarroll said ISU properly alerted federal research administrators, including officials of the federal Office of Research Integrity, and kept them informed of the internal investigation.

McCarroll also said that the remaining members of the research team have undertaken training in ethics. No one else on the team, including lead professor Michael Cho, has been accused of wrongdoing. ISU's investigation was closed after Han resigned.

Han signed a confession in September and agreed to be banned for five years from participating in research financed by the National Institutes of Health. The Register has been unable to reach him for comment.

Caplan said there have been a few criminal prosecutions for research misconduct involving trials of new drugs on people. But such prosecution is almost unheard of in cases involving animal research, he said.

The National Institutes of Health awarded the team $19 million in research grants over several years, including when the researchers used to work at Case Western University in Ohio. A federal

PHOTO GALLERIES

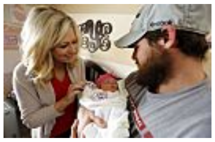
Nate, Laura and little Caralyn Yoho's story

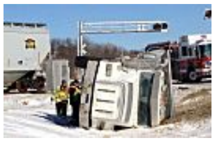
Train, truck accident in West Des Moines

SPONSORED LINKS Sponsored Content by Taboola

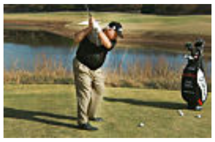
Classic golf mistake amateurs make at t…
Square to Square Method

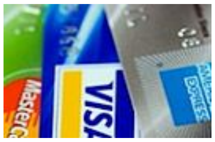
Experts Select The Best Credit Cards of…
Next Advisor

ADVERTISEMENT

ADVERTISEMENT

administrator said $10 million of the money came in response to the "exciting results" the researchers reported about their AIDS vaccine. About $4 million has yet to be spent. McCarroll, the ISU spokesman, said the university has not been asked to repay any money, and it has not been told that any of the remaining money would be withheld.

Ivan Oransky, a New York physician and journalist who writes about research ethics, said he would be surprised if federal officials try to recoup the grant money. "It's not a high priority for NIH to push for restitution because the money wouldn't go back to them. It would go back to the Treasury," he said.

Oransky said Han's banishment was unusually strong punishment. Few scientists even lose their jobs after being caught in misconduct, he said. Oransky agrees with Caplan that criminal prosecution should have been considered in the ISU case.

A National Institutes of Health spokeswoman said Friday that she could not reach officials in her agency to provide a comment.

Cho, the professor who leads the ISU team, has expressed disappointment in his former colleague, but he vowed to continue with vital work toward developing an AIDS vaccine. He did not respond Friday to a request for comment about whether law enforcement officers should have been notified of the situation.

A spokesman for U.S. Attorney Nick Klinefeldt of Des Moines said the office would not comment on the matter.

View Comments (7) | **Share your thoughts »**

TOP VIDEO PICKS
selected by Taboola

What you don't know about Mega Millions | U…
Dec 16, 2013

Inside Wrestling talks with ISU coach Kevin Jackson
Jan 7, 2014

YOU MIGHT BE INTERESTED IN

Zach Johnson gains win No. 11, explains why he's on a…

ISU football: Charge against David Irving is dropped

In this age of e-books, printed page still alive

Train flips dump truck into ditch in West Des Moines

Binge Viewing: Why almost everyone is a culprit these…

Whirlpool WRT359SFYB - Reviewed.com Refrigerators (Refrigerator Info)

SPONSORED LINKS

Aunt Gets Horrifying Christmas 'Present' From Nephew… (Articles in the News)

Alabama quarterback Luke Del Rio announces he's… (Sports Illustrated)

Vanessa Williams' Surprising DNA Test (Ancestry.com)

The 10 Worst Hotels and Motels in America (The Fiscal Times)

Officials Identify the Surprising Killer of 27 Bald… (Take Part)

11 Public Universities with the Worst Graduation Rates (The Fiscal Times)

[?]

Fiim: ISU's shooting woes

SPONSORED LINKS

Sponsored Content by Taboola

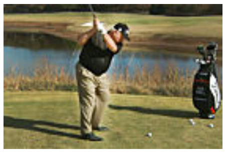

Classic golf mistake amateurs make at th…
Square to Square Method

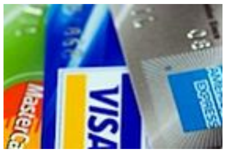

Experts Select The Best Credit Cards of …
Next Advisor

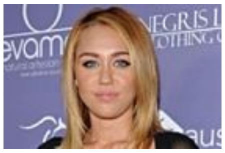

We Can't Help But Stare At Miley Cyrus
StyleBistro

ARCHIVES

View the last 30 days

Yesterday, Jan. 17
Thursday, Jan. 16
Wednesday, Jan. 15
Tuesday, Jan. 14
Monday, Jan. 13
Sunday, Jan. 12
Saturday, Jan. 11

See our paid archives for news older than a week.

juice

Latest stories

Shape up with geometric prints
Saturday YP essentials
Find college fun, cheap drinks at Peggy
Anatomy of a Drink: Apple cider

More Juice
Photo galleries
Newsletter
Calendar

PROMOTIONS

RAGBRAI
enter the 2014 BACON QUEEN contest
Embrace Iowa
R Earn extra income

CUSTOMER SERVICE

Place an ad
Subscribe Today!
Submit a milestone
Sign up for EZPay
View milestones
Manage Account
Work for us
Delivery problem
Feedback/news tips
Pay your bill
Newspapers in Education

ADVERTISEMENT

Site Map I Back to Top

NEWS
Politics
Crime & Courts
Education
Kyle Munson
Nation & World
Weather
Archive
Investigations & Watchdog Reports
Reader's Watchdog
Famous Iowans

OBITUARIES
Submit an obituary
Submit a memorial

SPORTS
HawkCentral
Cyclone Insider
High School Insider
Bryce Miller
UNI Panthers
Drake Bulldogs
National Sports
History & Databases
Photos

OPINION
Editorials
Rekha Basu
Kathie Obradovich
Guest Essays
Letters to the Editor
Your 2 Cents' Worth
Blogs
A Better Iowa

BUSINESS
Agriculture
Central Iowa Networking
Green Fields
Biz Buzz
Finance & Insurance
New in the Neighborhood
Iowa's Top Workplaces
Iowa Innovation
Shopping Scene

LIFE
Mike Kilen
Juice
Young Professionals
Health
Dating
Milestones

ENTERTAINMENT
Datebook
Events
Dining
Music
Theater
Movies
TV Listings
Comics

COMMUNITIES
Altoona
Ankeny
Clive
Des Moines
Indianola
Johnston
Norwalk
Urbandale
Waukee
West Des Moines

HELP
About us
Subscriber Solutions
Advertising Solutions
Reader Solutions
Register Media
Contact us

FOLLOW US
Twitter
Facebook
Mobile
RSS
E-mail Alerts

News | Jobs | Cars for Sale | Homes for Sale | Apartments for Rent | Shopping

GANNETT

Copyright © 2014 www.desmoinesregister.com. All rights reserved.
Users of this site agree to the Terms of Service, Privacy Notice/Your California Privacy Rights, and Ad Choices
